# Ecosystem Resilience of a South African Mesic Grassland with Change from Rotational to Continuous Grazing

**DOI:** 10.3390/d15121187

**Published:** 2023-11-29

**Authors:** Nomusa Chonco, Rob Slotow, Zivanai Tsvuura, Sindiso Nkuna

**Affiliations:** Centre for Functional Biodiversity, School of Life Sciences, University of KwaZulu-Natal, Pietermaritzburg 3201, South Africa

**Keywords:** forage quality, soil properties, mesic grassland

## Abstract

Grazing practices affect the soil and vegetation of grasslands, which further influence the provision of ecosystem services and the productivity of grasslands. We determined the ecosystem resilience of a mesic grassland under three grazing management systems in the Pakkies area, (30°33′08′′S, 29°25′22′′ E), South Africa: cooperative (continuously grazed since 2017), commercial (rotationally grazed for >20 years), and communal (continuously grazed for >20 years) farms. This was carried out by measuring the penetration resistance and infiltration, soil nutrients, forage quality contents for livestock, veld condition, plant species composition and richness, and functional diversity. The soils had a higher penetration resistance in the continuously grazed communal farm, while water infiltration was highest in the continuously grazed cooperative farm. The plant species and functional diversity were greater in the rotationally grazed commercial farm than in the continuously grazed communal and cooperative farms. The continuously grazed cooperative farm had the highest veld condition score (97%), while the rotationally grazed commercial and the continuously grazed communal farms had 82% and 56% veld condition scores, respectively. The forage quality and soil nutrients were generally similar among all farms. The lower plant diversity observed with continuous grazing may indicate that the ecological system was not as resilient concerning this type of grazing. However, for forage quality, soil nutrients and veld condition, continuous grazing was resilient, which indicates that rotational grazing may not be better than continuous grazing for livestock production in this specific region. As long as a minimum level of ecological resilience can be retained, continuous grazing can sustain effective animal production, particularly for small-holder farmers.

## 1 Introduction

Grasslands occupy at least 40% of the earth’s surface and are one of the most diverse ecosystems worldwide [1–3]. The mesic grasslands, defined as regions that receive greater than 600 mm of mean annual rainfall, provide essential ecosystem services [4]. They support large numbers of herbivores by providing forages and habitats to many animals, including insects and birds [5]. Mesic grasslands also regulate water flow due to the dense ground cover that ultimately decreases runoff and soil erosion, allowing water to infiltrate the soil [1]. Grazing by large mammals is one of the major disturbances influencing the ecological structure and community composition of these grasslands [6,7]. The impacts of grazing on plant species diversity differ among management practices, grazing intensity, and herbivore behaviour [8–10].

Grazing management practices are defined as various grazing systems that are used to manage and improve the productivity and plant diversity of grasslands. Grazing management systems are aimed at conserving the plant diversity, landscape, and soil of grassland [11,12]. While grazing management systems are intended to enhance the productivity of grasslands, their impacts on grassland ecosystem resilience and productivity have been largely ignored. Here, resilience is defined as the ability of a system to cope with a disturbance without changing to an alternative state and losing its functions and services [13,14]. Resilience at a local scale depends on the vegetation structure and the dynamics of a grassland that are driven by disturbances, such as fire and grazing pressure.

Continuous grazing consists of an open area in which animals can graze throughout the year. Under continuous grazing, domestic animals such as sheep, cattle, and horses can utilize the grassland freely without restrictions for extended periods [15,16]. However, selective grazing and overgrazing are the major problems associated with continuous grazing, as it purportedly reduces the plant species diversity and richness of grasslands [17,18]. Overgrazing may reduce plant basal cover, thereby increasing soil exposure, which leads to soil compaction [19]. The increase in bare soil increases runoff and may further cause soil loss and degradation [20]. Continuous grazing may result in an unbalanced biomass ratio because some species are avoided; thus, accumulate more unpalatable biomass while others are overgrazed [16]. Communal and cooperative farmers often use a continuous grazing system in their rangelands and do not apply structured management as to where or when grazing by livestock takes place [21].

In contrast, rotational grazing involves grazing camps, some of which are grazed, while others are rested [17,22]. In commercial farms, a rotational grazing system has prevailed, and it has been recommended as an effective management tool to maintain rangelands [11]. Rotational grazing allows for an even distribution of grazing and hence reduces selective grazing and overgrazing [23–25]. However, irrespective of the practical evidence obtained, there is an ongoing debate regarding the notion that rotational grazing results in better outcomes compared to continuous grazing [26,27].

Here, we determined the ecosystem resilience of a mesic grassland under three grazing management systems: cooperative (continuously grazed since 2017), commercial (rotationally grazed for >20 years), and communal (continuously grazed for >20 years) farms. The aim was to determine the sustainability of the ecological system and its productivity in terms of livestock production. The objectives were to determine the effects of grazing practices on: (1) the resilience of soil characteristics, such as soil physical properties (soil penetration resistance, water infiltration), and soil chemical characteristics (nutrient analysis); (2) the resilience of the factors that affect livestock production, which are forage quality contents for livestock and veld condition; and (3) the resilience of plant diversity by assessing the grassland plant species composition and plant functional diversity.

## 2 Materials and Methods

### 2.1 Study Sites

The study was conducted in the Pakkies village (30°33′08′′ S, 29°25′22′′ E; 1302 m above sea level) near Kokstad in the Harry Gwala District, KwaZulu-Natal Province, South Africa. The Pakkies area receives a mean annual rainfall of 747 mm, and the mean annual temperature is 15 °C. The summer season has mean daily minimum and maximum temperatures of 12 °C and 26 °C, respectively, in January, while winter has mean minimum and maximum daily temperatures of 2 °C and 22 °C, respectively, in July [28]. The vegetation is classified as the East Griqualand Grassland, and this area is part of the Maputaland–Pondoland–Albany biodiversity hotspot [29], which is an important centre of plant diversity and is one of the richest floristic regions in South Africa. The vegetation consists of grasses, herbs, herbaceous climbers, low shrubs, succulent shrubs, and trees with many endemics. The main grasses are *Themeda triandra, Hyparrhenia hirta*, and *Tristachya leucothrix*, whereas the dominant forbs are *Helichrysum odoratissimum, H. aureonitens*, and *Berkheya setifera*. The geology consists of mudstones and sandstones of the Beaufort Group and sedimentary rocks of the Molteno. These soils are clay-rich (clay content is 15–55%) and consist of Hutton, Clovelly, and Oatsdale soils, which form on sedimentary rocks, and Shortlands soil, which forms on dolerite [29].

### 2.2 Land Use History

The individual households from the village partake in crop (mainly maize) and live-stock (cattle, sheep, goats, and horses) farming, although goats and horses are less prevalent. The livestock graze continuously in the communal land that is specifically allocated for grazing. The communal rangeland (CF) has 412 ha of grazing land and has been grazed continuously for more than 20 years. An additional 350 ha is available on the cooperative farm (COP) owned by the community, with a small part of it used for crop production. The cooperative farm forms part of the area, which the Pakkies community successfully claimed from a commercial farm as part of the land reform programme. The COP farm has therefore been continuously grazed since 2017, and prior to that, it was rotationally grazed. A nearby commercial farm (CMF) is involved in cattle and sheep farming and has 430 ha of grazing land (Table 1). The CMF farm has been rotationally grazed for more than 20 years.

### 2.3 Data Collection

#### 2.3.1 Study Setup

The sampling sites in all three farms were similar in slope, with analogous abiotic and biotic characteristics, and away from watering points, as we wanted to control for environmental factors. Ten 10 m × 10 m plots were laid out on each farm, which were placed 10 m away from the fence to reduce edge effects and were separated from each other by 15 m. It was not feasible for this study to replicate treatments (farming systems) due to environmental heterogeneity at the landscape scale, which would interfere with the results [31,32]. Thus, pseudo-replication of the treatments is acknowledged as a potential problem in interpreting the results of this study (see [33,34]). Nevertheless, replicating the plots and quadrats, including spacing, allows one to surmise that plants measured in one plot are entirely independent of the plants measured in another plot.

#### 2.3.2 Soil Physical Properties

Penetration resistance was measured as an indicator for soil compaction using a dynamic cone penetrometer [35]. Each plot was divided diagonally. On five points of diagonals (3.5 m apart), soil penetration resistance (five measurements per each point) was measured, resulting in a total of 25 measurements per plot. For each reading, a dynamic cone with a hammer of 2 kg was released from a height of 60 cm until it touched the collar of the shaft. When the cone had entered the soil, depth measurements after each strike were recorded at five strikes per point and expressed as J m^−1^ [36].

Water infiltration was measured at the exact five points as above using a Decagon disk infiltrometer (Decagon Devices, Pullman, Washington State, United States of America). The readings were taken at 30 s intervals for 5 min at each point. We used a maximum starting volume of 80 mm. The slope of the cumulative infiltration curve and the values found from the cumulative curve were used to calculate the hydraulic conductivity of the soil using the equation:

K=C1/A

where *K* is the hydraulic conductivity, C1 is the slope of the curve of the cumulative infiltration versus the square root of time, and A is the parameter of a soil type, suction rate, and the radius of the infiltrometer (Decagon Devices, Pullman, Washington State, United States of America).

#### 2.3.3 Soil Chemical Properties

Five soil samples were collected from the 0–15 cm soil layer using a 55 mm diameter soil augur. The soil samples were collected from every second plot, with three subsamples randomly collected per plot to make one composite sample. The soil samples were air-dried, sieved (2 mm), labelled, and sent to the Soil Science Laboratory of the Provincial Department of Agriculture and Rural Development at Cedara. Soil pH (KCl) and extractable P were analysed using the Hunter method; soil N and organic C were determined using the automated Dumas dry combustion method [37]. Exchangeable cations such as Mg, Ca, and K and extractable Zn and Mn were analysed through atomic absorption [37].

#### 2.3.4.Forage Quality

We assessed the forage quality using the bulk sampling method, which is characterised by clipping aboveground parts of main grasses based on their abundance. Ten tufts of each of a palatable grass, a grass acceptable to herbivores, and an unpalatable grass from each of the farming systems were cut and put into four replicate brown sample bags per species, making a total of 12 samples per farm [38]. The sampled tufts (>5 m apart) were selected randomly from sampling points on each farm. The three grasses represent species that are palatable (*T. triandra*), acceptable (*T. leucothrix*), and unpalatable (*Elionurus muticus*) to grazers [39]. Grass samples were oven-dried (60 °C for 48 h) and ground to 1 mm particle size before analyses using acid detergent fibre (ADF), neutral detergent fibre (NDF), and crude protein (CP), outlined in the method of [37].

#### 2.3.5 Veld Condition Assessment

Basal cover and tuft size may influence runoff and soil erosion in grasslands [40,41]. A decrease in basal cover and small grass tufts are usually associated with a change in vegetation composition due to intense grazing [42], resulting in increased runoff and soil erosion [43]. We assessed basal cover and tuft size using the distance method for veld condition assessment, adapted from [44]. We randomly laid down a 100 m line transect, down the catena at each farming type, and at 1 m intervals along the transect, which was the distance from the point to the closest tuft; the diameter of the tuft for 100 points in each farm were measured. The identity of the plant species nearest to the point and its life form (grasses) were also recorded. We identified grasses at species level and arranged them according to their ecological group and their grazing value following [39].

#### 2.3.6 Plant Species Composition, Diversity, and Functional Diversity

In each 10 m × 10 m plot, a 0.5 m × 0.5 m quadrat was randomly placed 15 times to determine species composition and abundance of grasses and forbs. The plants rooted within the quadrat were identified to species level and recorded. For grasses, the percentage cover of species in the quadrat was visually estimated to the nearest 5%, and species composition was expressed as the mean cover percentage of each species per plot [45]. For forbs, the abundance of each species was quantified by counting the number of individual plants based on a cluster of stems rooted in each quadrat [46] and was expressed as mean density per plot. The life history of forb species, whether they were perennials or annuals, was recorded; the aboveground growth form was categorized as either cauline prostate, cauline erect, radical prostate, or radical erect based on [47,48]. Additionally, the underground growth forms of forbs were evaluated based on their root systems (root tubers, tap toots, woody tap roots, fibrous roots, bulbs, and rhizomes) following [48]. The forbs were identified at species level and classified according to their families. The proportion of each forb family’s contribution to the total species between the commercial farm, cooperative farm, and communal farm was calculated.

### 2.4 Statistical Analyses

We used a one-way analysis of variance in IBM SPSS Statistics 27 to test whether there were significant differences for soil physical and chemical properties, forage quality (NDF, ADF, and CP), plant species diversity, and basal cover among the commercial farm (rotational grazing), cooperative farm (continuous grazing), and communal farm (continuous grazing). The Kolmogorov–Smirnov normality assumption was satisfied (*p* > 0.05), and Levene’s test of equality of variances assumption was met. We used the multiple comparisons (Tukey) to test the differences between the farms.

Diversity was expressed as Shannon–Weiner diversity (*H*′), Pielou’s evenness (*J*) and species richness. Shannon–Weiner diversity index and Pielou’s evenness were calculated using the percentage cover of grasses and abundance of forbs separately per plot. Species richness was the total number of grass and forb species separately per plot.

The Jaccard index was used to calculate the percentage similarity of the species composition of grasses and forbs between pairs of farms based on the formula:

Cj=j/(a+b−j)

where j is the number of species found in both sites, a is the number of species in site A, and b is the number of species found in site B [49].

We performed a redundancy analysis (RDA) with Monte Carlo permutations′ test of significance in CANOCO 4.5 package [50] to test whether continuous or rotational grazing among commercial, cooperative, and communal farms accounted for the patterns of variation of species composition of grasses and forbs and the growth habits (aboveground and belowground habits) of forbs.

## 3 Results

### 3.1 The Effect of Livestock Grazing on Soil Physical and Chemical Properties, Forage Quality, and Veld Condition

**Figure 1:**
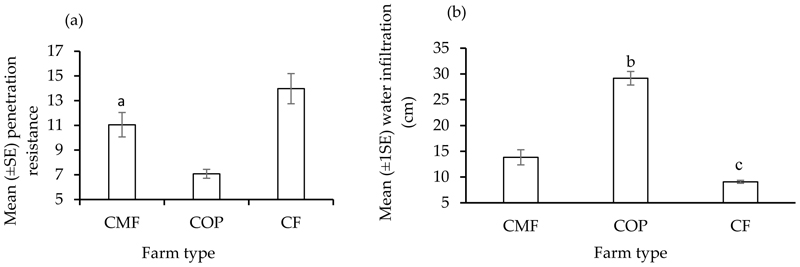
The mean (±SE) penetration resistance (**a**) and water infiltration (**b**) measured at the commercial (CMF), cooperative (COP), and communal (CF) farms at Pakkies near Kokstad in KwaZulu-Natal province, South Africa. Different superscript letters indicate significant (*p* < 0.05) differences among farms.

Grazing caused significantly greater penetration resistance in the continuously grazed CF than in the rotationally grazed CMF and continuously grazed COP farms. However, water infiltration was higher in the continuously grazed COP farm than the other two farms (Figure 1).

The mean concentration of soil N, Mg, Zn, and Ca was greater in the continuously grazed COP and CF farms than in the rotationally grazed farm. The soil organic carbon was greater in both the rotationally grazed CMF and continuously grazed COP farms and lower in the CF farm (Table 2).

The forage quality (ADF, NDF, and CP) of *Themeda triandra* and *Tristachya leucothrix* was similar between grazing practices and among the farms (Table 3). For *Elionurus muticus*, the amount of CP was significantly different between the grazing managements; the highest CP concentration was observed in the rotationally grazed CMF farm, followed by the COP farm, and the CF farm had the lowest concentration. Other parameters (ADF and NDF) were similar among the farms for this species.

The grazing systems did not have a significant effect on the herbaceous layer basal cover (*p* > 0.05) among farms. However, the continuously grazed COP farm had the highest veld condition score (97%), followed by the rotationally grazed CMF farm (82%) and then the continuously grazed CF farm (56%).

### 3.2 The Effect of Grazing on Plant Species Composition, Diversity, and Functional Diversity

A total of 26 grass species (Table S1) and 55 non-grass herbaceous species classified as forbs (Table S2) were recorded on all the farms. The Jaccard index showed that the rotationally grazed CMF farm and the continuously grazed COP farm were 52% similar with regard to grasses and 50% similar with regard to forbs. The CMF and CF farms were 43.4% similar with regard to grasses and 32.7% similar with regard to forbs. Between the CF and COP farms, the grasses were 57% similar, and the forbs were 37.8% similar.

**Figure 2:**
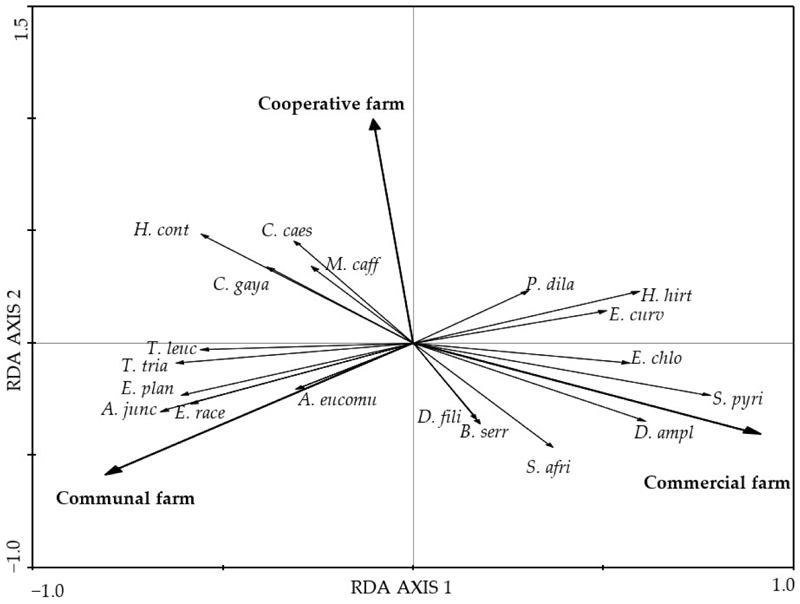
A standardized RDA biplot of the composition of grass species from the Pakkies area in Kokstad, KwaZulu-Natal province, South Africa. The species composition was measured from the commercial, cooperative, and communal farms and plotted on RDA axes 1 and 2 with eigenvalues of 0.332 and 0.071 representing 33.2% and 40.4% of the total variance, respectively. The species names are *T. leuc* = *Tristachya leucothrix, T. tria* = *Themeda triandra, E. plan* = *Eragrostis plana, A. junc* = *Aristida junciformis, E. race* = *Eragrostis racemosa, P. uvil* = *Paspalum urvillei, A. eucom* = *Andropogon eucomus, E. chlo* = *Eragrostis chloromelas, S. pyri* = *Sporobolus pyramidalis, D. ampl* = *Diheteropogon amplectens, S. afri* = *Sporobolus africanus, B. serr* = *Brachiaria serrata, D. fili* = *Diheropogon filifolius, H. cont* = *Heteropogon contortus, C. gaya* = *Chloris gayana, M. caff* = *Microchloa caffra, C. caes* = *Cymbopogon caesius, P. dila* = *Paspalum dilatatum, H. hir* = *Hyparrhenia hirta*, and *E. curv* = *Eragrostis curvula*.

**Figure 3:**
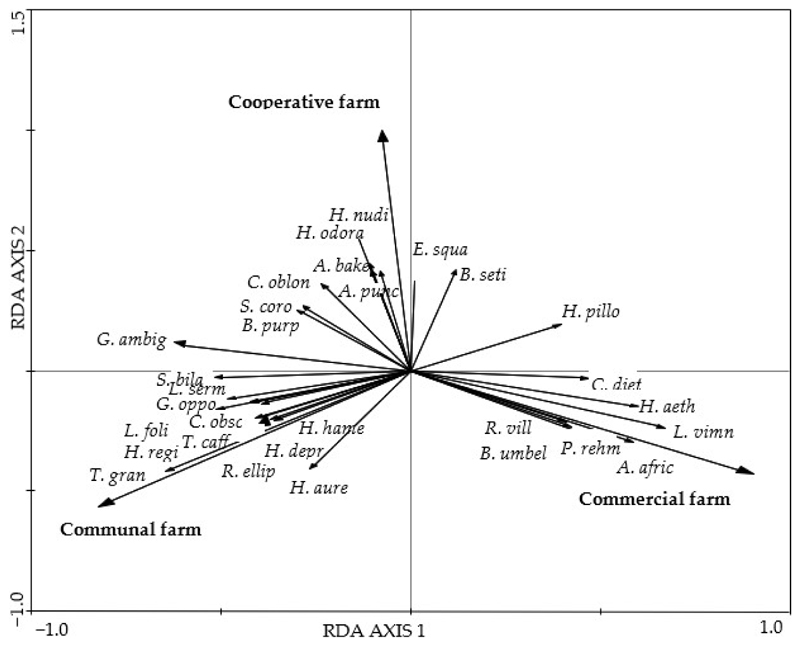
A standardized RDA biplot of the composition of forb species from the Pakkies area in Kokstad, KwaZulu-Natal province, South Africa. Species composition was measured from the commercial, cooperative, and communal farms and plotted on RDA axes 1 and 2 with eigenvalues of 0.140 and 0.078 representing 13% and 21.8% of the total variance, respectively. Species names are *S. bila* = *Striga bilabiata, L. serm* = *Launacea sermentosa, G. oppo* = *Gladiolus oppositiflorus, L. foli* = *Lotononis foliosa, C. obsc* = *Crotalaria obscura, H. rigi* = *Hypoxis rigidula, T. caff* = *Talinum caffram, H. hame* = *Hypoxis hemerocallidea, H. depr* = *Hermania depressa, H. aure* = *Helichrysum aureonitens, R. ellip* = *Rafnia elliptica, T. gran* = *Tephrosia grandiflora, C. diet* = *Cineraria dieterlenii, H. aeth* = *Hibiscus aethiopicus, L. vimn* = *Lotononis viminea, A. afric* = *Asparagus africanus, B. umbel* = *Berkheya umbellata, R. vill* = *Rhynchosia villosa, P. rehm* = *Polygala rehmannii, H. odora* = *Helichrysum odoratissimum, A. bake* = *Aster bakerianus, H. nudi* = *Helichrysum nudifolium, C. oblo* = *Cephalaria oblongifolia, S. coro* = *Senecio coronatus, B. purp* = *Berkheya purpurea, G. ambi* = *Gerbera ambigua, E. squa* = *Eriosema squarrosum, B. seti* = *Berkheya setifera, H. pilo* = *Helichrysum pilosellum*, and *A. punc* = *Acalypha punctata*.

There was a significant difference in the species composition of grasses and forbs among farm types (*p* < 0.01) in both cases based on the RDA first canonical axis (Figures 2 and 3). The grasses *Sporobolus pyramidalis* and *Diheteropogon amplectens* and the forbs *Lotononis viminea* and *Asparagus africanus* attained higher cover value percentages in the CMF farm than in the COP farm (Figure 2). The unpalatable perennial grasses *Cymbopogon caesius* and *Microchloa caffra* and the palatable perennial grass *Heteropogon contortus* attained higher cover value percentages than in the COP and CF farms. The forbs *Helichrysum odorassitimum, H. nudifolium*, and *Berkheya setifera* attained higher coverage value percentages in the COP farm. The grasses *Aristida junciformis* and *Eragrostis racemosa* and the forbs *Tephrosia grandiflora* and *Helichrysum aureonitens* attained higher cover value percentages in the CF farm than in the CMF and COP farms.

The species diversity (*H*′) and species richness of grasses and forbs were higher in the rotationally grazed CMF farm, followed by the continuously grazed COP farm and then the continuously grazed CF farm (Table 4). Species evenness (*J*) of grasses and forbs was similar among the farms (*p* > 0.05).

Overall, there were 19 plant families. Of these, the most abundant plant families were the Poaceae, Asteraceae Fabaceae, and Malvaceae. Grazing management may have affected the number of species found in each family among the three farms. For example, in the Malvaceae, we recorded one species in the continuously grazed COP farm, two species in the continuously grazed CF farm, and three species in the rotationally grazed CMF farm. Similarly, there were two species of Asparagaceae in the COP farm, none in the CF farm, and four species in the CMF farm (Table S3).

### 3.3 Growth Forms of Forbs above Ground and Below Ground

**Figure 4:**
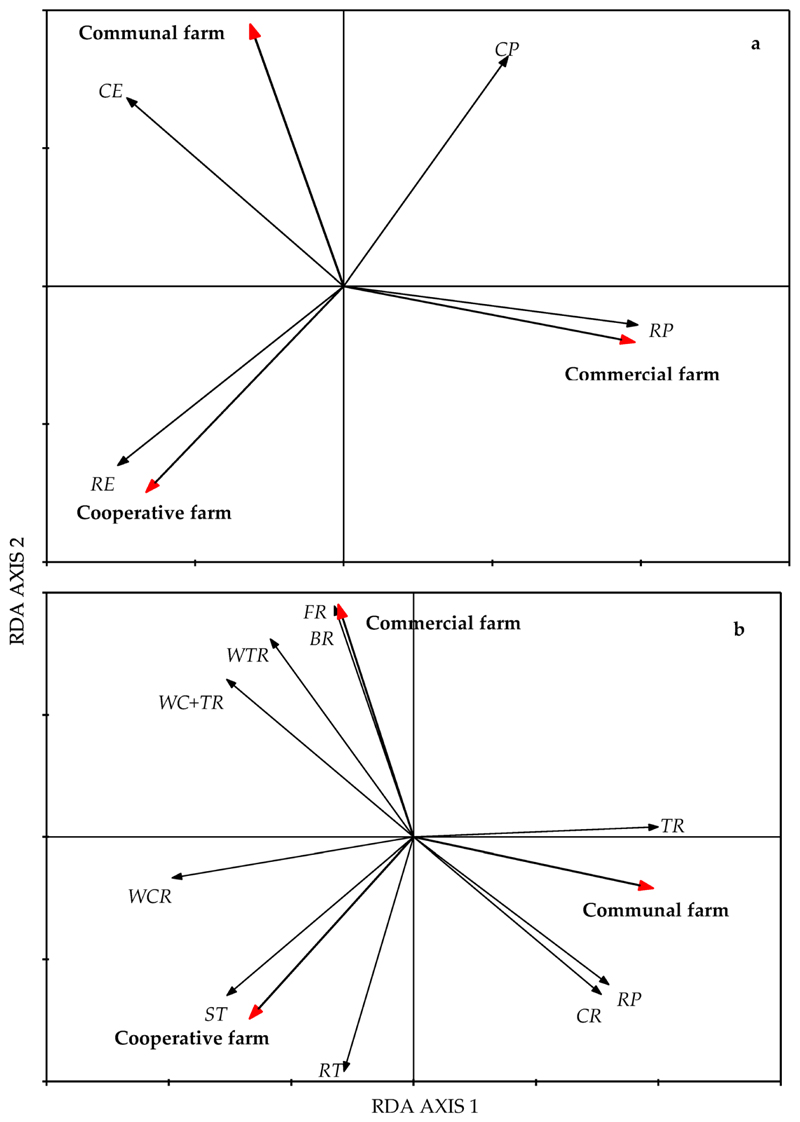
Biplot of growth forms of forbs (**a**) aboveground and (**b**) belowground based on a standardized RDA from the Pakkies area in Kokstad, KwaZulu-Natal province, South Africa. Aboveground form categories were RE = radical erect, RP = radical prostrate, CE = cauline erect, and CP = cauline prostrate. Belowground form categories were TR = taproots, ST = stem tubers, WCR = woody cauline roots, RT = root tubers, RP = root parasite, CR = corm, BR = bulb, WC + TR = woody caudex with taproots, FR = fibrous roots, and WTR = woody taproots.

The overall aboveground growth form (AGF) and belowground growth form (BGF) of forbs significantly differed among the farms (*p* < 0.01) based on the first canonical axis (Figure 4). Axis 1 accounted for 61.4% of the variation for the AGF and 79.4% for the BGF. The CMF farm was dominated by forbs with radical and cauline prostrate growth forms. In contrast, the COP farm was dominated by radical erect forbs, while the CF farm was dominated by cauline prostrate and cauline erect forbs. The forbs with woody taproots, bulbs, and fibrous roots were more abundant on the CMF farm, while stem tubers, root tubers, and woody caudex roots were more abundant on the COP farm. Taproots, root parasites, and corms were abundant in the CF farm (Figure 4).

## 4 Discussion

### 4.1 Effects of Grazing Systems on Soil Physical and Chemical Properties

We found that the soils had a higher soil penetration resistance in the continuously grazed grassland in the CF farm. As a result, water infiltration was also lower in the CF farm due to high penetration resistance in the soil. This may be explained by a high stocking rate (not assessed in this study) due to continuous grazing (not assessed in this study) in the CF rangelands, which, consequently, caused soil compaction [51]. However, water infiltration was noticeably higher in the continuously grazed COP farm than in the other farms, and again, that could be the result of varying stocking rates in the COP and CF farms irrespective of the same grazing management.

The amount of soil organic C (OC) was higher in the CMF and COP farms than in the CF farm. The lower OC in the continuously grazed CF farm may be influenced by the trampling of aboveground vegetation, which caused depletion of organic matter and litter [21]. Another possible explanation for a low soil OC is the pronounced penetration resistance (soil compaction) in the communal area, which may have negatively influenced not only the erosion of organic matter due to lower water infiltration [51] but also the aboveground vegetation growth and its OC input [52]. Similar results were found by [53] in which there was a decline in soil organic matter content in the grazing lands, which was attributed to overgrazing and the compaction of the soil. The higher levels of available soil N content in the CF and COP farms could be attributed to the high stocking density of livestock and their excreta, which accumulate soil nutrients.

### 4.2 Effects of Livestock Grazing on Forage Quality

The nutritional value and forage quality of grasses are closely linked to the type of grazing management [54]. In this study, we found no effect of grazing management practice among the farming systems on the amount of NDF, ADF, and CP in the key foraging species *Themeda triandra* (a decreaser) and *Tristachya leucothrix* (an increaser I) [55]. These results are consistent with [56] in which the authors found similar amounts of fibre (NDF and ADF) and CP under continuous grazing, rotational grazing, and mob grazing on the temperate grasslands of central Virginia. The authors of [57] also found no significant differences in the nutritive value of NDF and CP of ryegrass between rotational and continuous grazing. However, the CP content of an increaser III species, such as *Elionurus muticus*, was significantly different among the grazing systems. The difference in the CP content could not have been associated with the grazing management but may have been associated with the growing stages of *E. muticus*, although the CP content was higher in the rotationally grazed CMF farm than in the COP and CF farms. One would assume that selective grazing for species other than *E. muticus* (due to its unpalatability) in the CF and COP farms could have caused it to grow and mature, thus causing it to lose its quality more than in the CMF farm.Among the factors that influence forage quality, the stage of maturity of the plants stands out as a primary factor that influences nutritional quality [58]. As a plant matures, its nutritional value declines due to increasing lignin or decreasing crude protein content [27,59].

The COP and CF farms had a comparable basal cover that is also greater than 12%, which is considered a characteristic of good management of the rangeland. In addition, the veld condition assessment revealed that the COP farm had the greatest score and had the highest percentage of decreaser species such as *Themeda triandra* and *Brachiaria serrata*. This suggests that the grassland is in good condition and dominated by palatable grasses that provide quality forage for livestock. The CMF farm had the second-highest percentage of decreaser species, which also implies a grassland in good condition. The CF farm had the lowest veld condition score and was mainly dominated by increaser II and III species, such as *Hyparrhenia hirta* and *Heteropogon contortus*. The high abundance of increaser species in the CF farm may be explained by high grazing pressures, a severe overutilization of the veld, and selective grazing, which resulted in the replacement of decreaser species by increaser species [60].

### 4.3 Effects of Livestock Grazing on Plant Species Diversity and Functional Diversity

Grazing affects plant species diversity and richness by either causing a loss of species to or a colonisation of species by the environment [22]. A decrease in species composition may reflect a negative response of plants to grazing [11], indicating a non-resilient ecosystem at a community level characterised by a reduced community composition [61]. Consistent with other research, e.g., [7,22,62], this study showed that grazing systems affected the species diversity and species richness of grasses and forbs. The species diversity and species richness of grasses and forbs in the continuously grazed COP farm were markedly lower than that of the rotationally grazed CMF farm but higher than that of the continuously grazed CF farm. Moreover, the similarity index showed the turnover of grass and forb species between the CMF and COP farms and between the CMF and CF farms, with a lower number of shared species. A decrease in species diversity and species richness may cause grazing-tolerant species to replace grazing-intolerant species [3]. This could be attributed to selective grazing, where livestock only graze on the palatable grasses. Additionally, this can further be explained as a non-resilient ecosystem at the community level where there is a loss or change in species diversity due to disturbances [63], specifically grazing management in this study.

Consistent with other research, the Asteraceae, Fabaceae, and Malvaceae were the most dominant forbs in the three farming systems. This pattern could be explained by dispersal qualities and resource attainment in the Asteraceae and Fabaceae, respectively, thereby making them resilient to various grazing intensities [64]. However, the Fabaceae, Euphorbiaceae, Hypoxidaceae, and Scrophulariaceae were more common under continuously grazed grasslands in the COP and CF farms compared to the CMF farm. The Fabaceae was one of the most dominant families in heavy grazing plots in southwestern Madagascar [65]. They stated that legumes had a high capacity for regeneration and were resistant to trampling by animals.

The high abundance of unpalatable perennial grasses such as *Microchloa caffra, Cymbopogon caesius*, and *Chloris gayana* in the COP farm could be attributed to selective grazing and localised grazing patterns [19] because such species are more common in grasslands that are overgrazed. However, this was not detected in the veld condition assessment. Selective grazing mainly affects the persistence of annual grasses, causing changes in the structure and composition of species in each community, thereby influencing the stability of the ecosystem [66]. This supports the high abundance of perennial grasses in the COP and CF farms in this study and the notion that grazing may have affected the persistence of annual grasses whose abundance then decreased.

### 4.4 Functional Diversity of Forbs

The forbs in mesic grasslands are mostly perennials characterised by underground storage organs that facilitate sprouting ability. In this study, approximately 70% of the forbs were perennial bud-producing forbs classified by their underground storage organs [67]. The rotationally grazed CMF farm contained various forbs that were dominated by species with resprouting underground storage organs such as bulbs, woody taproots, and woody caudex taproots. The continuously grazed COP farm was dominated by forbs with resprouting underground storage organs, e.g., stem tubers, root tubers, and woody caudexes. On the contrary, the belowground growth form dominant in the CF farm was non-sprouting taproots and root parasites. The prevalence of forbs with the non-sprouting taproots and root parasites may be attributed to the absence of fire, which is known to facilitate the growth of these forbs [68].

The life history traits or the aboveground growth life forms are essential physiological characteristics of plants used to predict the response or tolerance of the plants to disturbances [48,69]. Prostrate forbs are generally abundant in continuously grazed grasslands where there is overgrazing and selective grazing, and they appear to be grazing resistant or not susceptible to heavy grazing [48,70]. Studies conducted in the mesic grasslands found that forbs with prostrate growth forms were relatively insensitive to grazing compared to the forbs with erect growth forms [45,71]. In this study the prostrate forbs were more abundant in the CMF farm, which practices rotational grazing, than in the continuously grazed COP and CF farms. The increase in forbs with an erect growth form on the COP and CF farms, which are continuously grazed, could be an indication that the continuously grazed COP and CF farms were not overgrazed.

## 5 Conclusions

We showed that continuous grazing reduced the species composition, diversity, species richness, and functional diversity of grasses and forbs. However, we observed no effects of rotational and continuous grazing on livestock production factors, such as forage quality, veld condition, and some of the soil chemical properties. Although there was a decline in soil C in the continuously grazed farms, our findings are not compelling enough to conclude that this was due to degradation in the continuous grazing systems. We did not particularly focus on the stocking rate and its effect on vegetation and soils. Therefore, it may be relevant for future studies to include the stocking rate to clarify the extent to which grazing management practices affect the composition and diversity of plant species, soil nutrients, and the forage quality of rangelands.

## Supplementary Material

Table S1

## Figures and Tables

**Table 1:** Details of grazing management for commercial (CMF), cooperative (COP), and communal (CF) farms at Kokstad, KwaZulu-Natal province, South Africa.

	CMF	COP	CF
Total size of grazing area (ha)	430	350	412
Mean herd size (AU) *	160	105	250
Number of paddocks	6	1	None
Mean paddock size (ha)	70	350	None
Mean period of occupation (days)	150	Throughout the year	Throughout the year
Mean period of absence (days)	90	-	-

* One AU is equal to one cow weighing 450 kg which gains 0.5 kg per day on forage with a digestible energy percentage of 55% [30]

**Table 2:** Soil chemical properties measured at the commercial (CMF), cooperative (COP), and communal (CF) farms in the Pakkies area, Kokstad in KwaZulu-Natal province, South Africa. Values are mean ± SE based on n = 5 and df = 2. Values in bold are significant at *p* < 0.05. Different letters indicate significant differences among the farms for each soil chemical property.

Soil Chemical Properties	CMF	COP	CF	F	*p*
P (mg/kg)	7.79 ± 1.81	9.26 ± 0.85	9.87 ± 0.42	0.814	0.466
K (mg/kg)	222.01 ± 31.66	264.51	237.97 ± 26.07	0.401	0.678
Mg (mg/kg)	194.31 ± 44.73 ^a^	314.83 ± 12.17 ^b^	400.35 ± 18.57 ^b^	12.89	**0.001**
Ca (mg/kg)	764.07 ± 116.66 ^a^	1118.54 ± 128.1 ^b^	1362.76 ± 84.4 ^b^	7.321	**0.008**
Soil pH (KCl)	5.00 ± 0.14	5.00 ± 0.09	5.00 ± 0.05	0.03	0.997
Zn (mg/kg)	1.52 ± 0.50 ^a^	2.41 ± 0.36 ^b^	3.88 ± 0.61 ^c^	6.17	**0.014**
Mn (mg/kg)	77.66 ± 13.44	121.18 ± 23.36	117.34 ± 13.42	1.92	0.189
Cu (mg/kg)	4.38 ± 0.07 ^a^	3.63 ± 0.15 ^b^	3.39 ± 0.26 ^b^	8.44	**0.005**
C (%)	3.32 ± 0.26 ^a^	2.94 ± 0.29 ^a^	1.34 ± 0.31 ^b^	8.07	**0.006**
N (%)	0.12 ± 0.04 ^a^	0.21 ± 0.02 ^b^	0.25 ± 0.02 ^b^	6.52	**0.012**

**Table 3:** Determination of forage quality of *Themeda triandra, Tristachya leucothrix* and *Elionurus muticus* from the commercial (CMF), cooperative (COP), and communal (CF) farms at Pakkies near Kokstad in KwaZulu-Natal province, South Africa using acid detergent fibre (ADF), neutral detergent fibre (NDF), and crude protein (CP). Values are mean (±SE). Values in bold are significant at *p* < 0.05. Different letters indicate significant differences between the farms.

Forage Quality	CMF	COP	CF	F	*p*
		*T. triandra*			
ADF (%)	43.99 ± 0.61	43.38 ± 0.53	45.89 ± 1.99	1.1	0.392
NDF (%)	77.81 ± 0.04	78.20 ± 0.53	82.05 ± 1.94	4.05	0.077
CP (%)	4.29 ± 0.29	5.02 ± 0.62	5.57 ± 0.68	1.31	0.337
		*T. leucothrix*			
ADF (%)	40.87 ± 2.79	42.43 ± 1.49	40.19 ± 0.18	0.394	0.69
NDF (%)	75.77 ± 1.59	79.77 ± 2.04	75.5 ± 0.17	2.541	0.159
CP (%)	7.35 ± 0.49	7.47 ± 0.42	6.5 ± 0.03	1.946	0.223
		*E. muticus*			
ADF (%)	40.37 ± 1.45	38.83 ± 0.38	40.06 ± 0.48	0.804	0.49
NDF (%)	74.41 ± 2.40	75.26 ± 0.39	78.20 ± 0.53	1.921	0.229
CP (%)	8.19 ± 0.11 ^a^	6.49 ± 0.08 ^b^	5.03 ± 0.62 ^c^	18.87	**0.003**

**Table 4:** Mean (±SE) Shannon–Weiner diversity index (*H*′), Pielou’s evenness index (*J*), and species richness (df = 2) of grass and forb species measured in commercial (CMF), cooperative (COP), and communal (CF) farms near Kokstad in KwaZulu-Natal province, South Africa. Values indicated in bold are significant (*p* < 0.05). Diversity values are based on *n* = 10 plots per farm. Different letters indicate significant differences between the farms.

	CMF	COP	CF	*F*	*p*
Grasses					
Diversity *H′* (m^−2^)	2.30 ± 0.05 ^a^	2.10 ± 0.06 ^b^	1.80 ± 0.09 ^c^	14.374	**<0.0001**
Evenness *J* (m^−2^)	0.92 ± 0.02	0.91 ± 0.01	0.88 ± 0.01	1.65	0.210
Richness (m^−2^)	12.20 ± 0.55 ^a^	10.20 ± 0.51 ^b^	7.90 ± 0.59 ^c^	15.22	**<0.0001**
Forbs					
Diversity *H*′ (m^−2^)	2.55 ± 0.02 ^a^	2.38 ± 0.03 ^b^	2.22 ± 0.02 ^c^	42.21	<**0.0001**
Evenness *J* (m^−2^)	0.95 ± 0.01	0.93 ± 0.01	0.93 ± 0.01	3.26	0.054
Richness (m^−2^)	14.80 ± 0.39 ^a^	12.90 ± 0.43 ^b^	10.90 ± 0.28 ^c^	27.46	**<0.0001**

## Data Availability

The data that support the findings of this study are available on request from the corresponding author.
