# Supplementary material for: Ecosystem Resilience of a South African Mesic Grassland with Change from Rotational to Continuous Grazing
Source: Diversity (Basel). Author manuscript; Available in PMC 2024 Jul 12. (PMC7616199; doi:10.3390/d15121187)
Supplement: Table S1 [file EMS197303-supplement-Table_S1.pdf]

diversity

MDPI

Supplementary information

Ecosystem Resilience of a South African Mesic Grassland with Change from Rotational to Continuous Grazing

Nomusa Chonco *, Rob Slotow, Zivanai Tsvuura and Sindiso Nkuna *

Centre for Functional Biodiversity, School of Life Sciences, University of KwaZulu-Natal, Pietermaritzburg 3201, South Africa; slotow@ukzn.ac.za (R.S.); tsvuuraz@ukzn.ac.za (Z.T.)
* Correspondence: nomsachonco18@gmail.com (N.C.); chamane@ukzn.ac.za (S.N.)

Table S1. The list of grasses (Poaceae) found in the commercial farm (CMF), cooperative farm (COP) and communal farm (CM) at Pakkies, near Kokstad, KwaZulu-Natal, South Africa. All the grasses are perennial. Nomenclature follows Fish et al. (2015) [72].

Species name	Ecological status	Grazing value
Alloteropsis semialata (R.Br.) Hitchc.	Increaser II	Moderate
Aristida junciformis Trin. & Rupr.	Increaser III	Low
Bothriochloa inscultpa (A.Rich.) A.Cumus	Increaser II	Moderate
Brachiaria serrata (Thunb.) Stapf	Decreaser	Moderate
Cymbopogon caesius (Hook. & Arn.) Stapf	Increaser I	Low
Digitaria monodactyla (Nees) Stapf	Increaser II	Moderate
Diheteropogon amplectens (Nees) Clayton	Decreaser	Moderate
Diheteropogon filifolius (Nees) Clayton	Increaser II	Low
Elionurus muticus (Spreng.) Kuntze	Increaser III	Low
Eragrostis capensis (Thunb.) Trin.	Increaser II	Moderate
Eragrostis chloromelas Steud	Increaser II	Low
Eragrostis curvula (Shrad.) Nees	Increaser II	Moderate
Eragrostis plana Nees	Increaser II	Low
Eragrostis racemosa (Thunb.) Steud.	Increaser II	Moderate
Harpochloa falx (L.f.) Kuntze	Increaser II	Low
Heteropogon contortus (L.) Roem. & Scholt.	Increaser II	High
Hyparrhenia hirta (L.) Stapf	Increaser II	Moderate
Microchloa caffra Nees	Increaser II	Low
Paspalum dilatatum Poir.	Increaser II	High
Paspalum urvillei Steud.	Increaser I	Moderate
Setaria sphacelata (Schumach.) Stapf. & C.E.Hubb. ex M.B.Moss	Increaser II	High
Sporobolus africanus (Poir.) Robyns. & Tournay	Increaser II	Low
Sporobolus_pyramidalis P.Beauv.	Increaser II	Low
Themeda triandra Forssk.	Decreaser	High
Trachypogon spicatus (L.f.) Kuntze	Increaser I	Low
Tristachya leucothrix Trin. ex Nees	Increaser I	Moderate

Table S2. The list of non-grass herbaceous plants found in the commercial farm (CMF), cooperative farm (COP) and communal farm (CM) at Pakkies area, near Kokstad, KwaZulu-Natal, South Africa. All plants are perennial. Nomenclature follows World Flora online.

Species name	Family	Life form
Acalypha punctata Meisn. ex Krauss	Euphorbiaceae	Herb
Ajuga ophrydis Burch. ex Benth.	Lamiaceae	Herb
Asparagus aethiopicus L.	Asparagaceae	Shrublet
Asparagus africanus Lam.	Asparagaceae	Herb
Aster bakerianus Burtt Davy ex C.A.Sm.	Asteraceae	Herb

Berkheya purpurea (DC.) Mast.	Asteraceae	Herb
Berkheya setifera DC.	Asteraceae	Herb
Berkheya umbellata DC.	Asteraceae	Herb
Centella asiatica (L.) Urb.	Apiaceae	Herb
Cephalaria oblongifolia (Kuntze) Szabó	Dipsacaceae	Herb
Cephalaria pungens Szabó	Dipsacaceae	Herb
Cineraria dieterlenii E.Phillips	Asteraceae	Herb
Crabbea acaulis N.E.Br.	Acanthaceae	Herb
Crassula lanceolata Endl.	Crassulaceae	Herb
Crotalaria obscura DC.	Fabaceae	Herb
Eriosema cordatum E.Mey.	Fabaceae	Herb
Eriosema distinctum N.E.Br.	Fabaceae	Herb
Eriosema squarrosum (Thunb.) Walp.	Fabaceae	Herb
Felicia wrightii Hilliard & B.L.Burtt	Asteraceae	Herb
Gerbera ambigua Sch.Bip.	Asteraceae	Herb
Gerbera piloselloides Cass.	Asteraceae	Herb
Gladiolus oppositiflorus Herb.	Iridaceae	Herb
Helichrysum aureonitens Broth. & Paris	Asteraceae	Herb
Helichrysum cymosum (L.) D.Don ex G.Don	Asteraceae	Herb
Helichrysum glomeratum Kirk	Asteraceae	Herb
Helichrysum nudifolium (L.) Less.	Asteraceae	Herb
Helichrysum odoratissimum Sweet	Asteraceae	Herb
Helichrysum pilosellum Less.	Asteraceae	Herb
Helichrysum splendidum Less.	Asteraceae	Herb
Hermannia depressa N.E.Br.	Malvaceae	Herb
Hibiscus aethiopicus L.	Malvaceae	Herb
Hibiscus pusillus Thunb.	Malvacea	Herb
Hypoxis argentea Harv. ex Baker	Hypoxidaceae	Herb
Hypoxis hemerocallidea Fisch., C.A.Mey. & Avé-Lall.	Hypoxidaceae	Herb
Hypoxis rigidula Baker	Hypoxidaceae	Herb
Launaea sarmentosa (Willd.) Kuntze	Asteraceae	Herb
Ledebouria cooperi (Hook.f.) Jessop	Asparagaceae	Bulb
Ledebouria ovatifolia (Baker) Jessop	Asparagaceae	Bulb
Lotononis foliosa Bolus	Fabaceae	Herb
Lotononis viminea (E.Mey.) B.-E.van Wyk	Fabaceae	Herb
Oxalis obliquifolia Steud. ex A.Rich.	Oxalidaceae	Herb
Pentanisia angustifolia Hochst.	Rubiaceae	Herb
Polygala rehmannii Chodat	Polygalaceae	Herb
Rafnia elliptica Thunb.	Fabaceae	Shrublet
Rhynchosia cooperi (Baker f.) Harv. ex Burtt Davy	Fabaceae	Herb
Rhynchosia minima (L.) DC.	Fabaceae	Shrublet
Rhynchosia totta (Thunb.) DC.	Fabaceae	Herb
Rhynchosia villosa (Meisn.) Druce	Fabaceae	Herb
Roella glomerata A.DC.	Campanulaceae	Shrublet
Senecio coronatus Harv.	Asteraceae	Herb
Senecio latifolius Mast.	Asteraceae	Herb
Senecio speciosus Willd.	Asteraceae	Herb
Striga bilabiata (Thunb.) Kuntze	Scrophulariaceae	Herb
Talinum caffrum (Thunb.) Eckl. & Zeyh.	Portulacaceae	Herb
Tephrosia grandiflora (L'Hér.) Pers.	Fabaceae	Shrublet

Table S3. Number of forb species for each family in the commercial (CMF), cooperative (COP) and communal (CF) farms at Pakkies near Kokstad, KwaZulu-Natal, South Africa.

Family	CMF	COP	CF
Acanthaceae	1	0	0

Apiaceae	1	1	1
Asparagaceae	4	2	0
Asteraceae	17	15	13
Campanulaceae	1	0	0
Caprifoliaceae	1	1	1
Crassulaceae	1	0	2
Dipsacaceae	0	0	1
Euphorbiaceae	8	6	11
Fabaceae	1	3	2
Hypoxidaceae	1	1	1
Iridaceae	1	1	1
Lamiaceae	3	1	2
Malvaceae	1	1	1
Oxalidaceae	1	0	0
Polygalaceae	0	0	1
Portulacaceae	1	0	0
Rubiaceae	0	1	1
Scrophulariaceae	1	0	0

References

72. Fish L., Mashau A.C., Moeaha M.J. and Nembudani M.T. 2015. Identification guide to Southern African grasses. An identification manual with keys, descriptions and distributions. Strelitzia 36. South African Biodiversity Institute, Pretoria. World Flora Online, http://www.worldfloraonline.org/, accessed on 19 November 2023.

Disclaimer/Publisher's Note: The statements, opinions and data contained in all publications are solely those of the individual author(s) and contributor(s) and not of MDPI and/or the editor(s). MDPI and/or the editor(s) disclaim responsibility for any injury to people or property resulting from any ideas, methods, instructions or products referred to in the content.
